# Implications of Aspirin for Melanoma Treatment: A Short Perspective

**Published:** 2017-08-17

**Authors:** Anita Thyagarajan, Ravi P Sahu

**Affiliations:** Department of Pharmacology and Toxicology, Boonshoft School of Medicine at Wright State University, Dayton, OH, USA

**Keywords:** Aspirin, Melanoma, PAF-R, SOX-2

## Abstract

Several human cancers including melanoma exhibit increased expression of inflammatory cyclooxygenases (COX) enzymes that catalyze the conversion of arachidonic acid to prostaglandins (PGs) implicated in tumor growth. As aspirin has been used in the treatment of various ailments including inflammatory diseases, and cancers due to its anti-inflammatory property via inhibiting COX enzymes its significance particularly in reducing the risk of advanced stage or metastatic melanoma has yielded mixed responses. This mini review addresses some of the discrepancies of implications of aspirin from preclinical and clinical studies, and recent updates into its mechanisms of actions in melanoma treatment.

## Introduction

Despite various available treatment options, the prognosis of melanoma remains grim [1]. This indicates the involvement and cross talks between several oncogenic signaling pathways including the driver mutations in central BRAF and NRAS genes leading to increased heterogeneity and resistance of melanoma tumors to targeted therapies [2]. Among other signaling cascades, activation of a G-protein coupled receptor in response to oxidized phospholipid mediator, platelet-activation factor (PAF) produced by several pro-oxidative stressors generating reactive oxygen species (ROS) plays important roles in favoring the growth of pre-existing melanoma tumors in preclinical studies [3–14]. Importantly, studies including ours have demonstrated that PAF and PAF-like species possess immunosuppressive properties and induce systemic immunosuppression that results in an augmentation of subcutaneously implanted murine B16F10 melanoma tumors growth via increasing regulatory T-cells (Tregs) in tumor microenvironment [6,10,11]. Notably, we have shown that therapeutic agents including cancer chemotherapy generate PAF agonists as a byproduct that augment the growth, and impede their efficacies in a PAF-receptor (PAF-R) dependent manner in experimental murine melanoma models [7,8]. Of significance, increased levels of PAF or PAF-R activity were detected in melanoma patients undergoing therapeutic treatments [7,8]. As melanomas express COX-2 enzyme which is the downstream target of PAF-R signaling pathway, we have shown that COX2 inhibition blocked PAF-R mediated effects of pro-oxidative stressors including cancer therapies in preclinical studies [3,7–9]. These findings indicate crucial roles of PAF-R signaling in melanoma tumorigenesis and melanoma therapies.

## Aspirin and Melanoma Treatment

Aspirin (acetylsalicylic acid) has long been used in the treatment of inflammatory diseases and possess anti-carcinogenic properties due to its ability to target COX enzymes and inhibit prostaglandins (PGs) synthesis [15]. Importantly, in clinical studies, while aspirin intake has been shown to reduce the risk of human cancers including gastric and colon cancer, there have been mixed responses regarding the use of aspirin and prevention of skin cancer including melanoma risk [16–18]. The findings suggested the need of more well-designed randomized controlled trials from large cohort to have more conclusive responses of aspirin intake in reducing the risk of advanced melanoma. This could also indicate the lack of detailed mechanistic studies of aspirin in preclinical melanoma models that resembles the highly aggressive and advanced stage of melanoma in humans. Notably, most preclinical studies in defining the role of aspirin in melanoma treatment have used the non-metastatic and less aggressive form of syngeneic B16F0 or B16F1 murine melanoma cells [19–22]. These findings indicated that aspirin treatment reduces the growth of melanoma cells in *in vitro* and *in vivo* models via various mechanisms and modulating distinct signaling cascades [19–22]. To answer this important concern, and investigate effects of aspirin against aggressive and metastatic melanoma model, we have recently taken advantage of highly aggressive and metastatic murine B16F10 syngeneic melanoma cells. Our studies demonstrated that aspirin treatment suppresses the growth of *in vitro* melanoma cells via reducing its survival in a dose and time dependent manner and inducing apoptosis [23]. However, presence of functional PAF-R in melanoma cells did not modulate aspirin sensitivity or effectiveness. Our *in vivo* findings confirmed the *in vitro* data that systemic intake of aspirin in drinking water ad libitum significantly reduced the growth of both PAF-R positive (B16-PAFR) and negative (B16-MSCV) melanoma tumors, and that the rates of tumor growth suppression were similar in PAF-R-expressing wild type (WT) and deficient (PAF-R−/−) mice [23]. Moreover, while aspirin treatment bypasses the PAF-R signaling, we investigated that aspirin targets prostaglandin F2 alpha (PGF2α) and Sry-related high-mobility Box-2 (SOX-2) gene to inhibit the *in vivo* growth of B16F10 melanoma tumors [23]. Interestingly, the expression of SOX-2 gene has been identified in several cancer models including melanoma, and linked in inducing tumor resistance or anti-apoptotic responses to standard therapies against cancers [24–29]. Importantly, exogenous treatment of PGF2α agonists and overexpression of SOX-2 by fibroblast growth factor 1 (FGF-1) significantly blocked aspirin-induced inhibition of melanoma cell survival and increased apoptosis [23].

## Conclusion

As the role of aspirin in modulating SOX2 expression in melanoma model was not been studied before, ours was the first report demonstrating the novel mechanism of action of aspirin in a highly aggressive murine melanoma model via SOX2-dependent-PAF-R-independent pathway. These studies further set forward the rationale of exploring this pathway for melanoma chemoprevention.

## Abbreviations:

PAF: Platelet-activating factor
PAFR: PAF-receptor
ROS: Reactive oxygen species
PGF2a: Prostaglandin F2 alpha
SOX2: Sry-related high-mobility box 2
COX: Cyclooxygenase
